# Boron-based fire retardancy for natural polymeric materials

**DOI:** 10.3389/fchem.2026.1777619

**Published:** 2026-02-19

**Authors:** Xuan Wang, Sheldon Q. Shi

**Affiliations:** Mechanical Engineering Department, University of North Texas, Denton, TX, United States

**Keywords:** bio-composites, boron-based fire retardancy, char formation, lignocellulose, natural polymers

## Abstract

The shift from fossil resources to natural polymers as the building blocks of a global bioeconomy is hampered by the intrinsic flammability of these bio-derived materials. In this paper, the recent advances in boron-based fire retardancy of natural materials are reviewed, highlighting the transition from macro-scale salt impregnation to molecular-level engineering of boron chemistry. Boron compounds act as a dual Lewis acid catalyst for dehydration and subsequent char formation, and as a glassy physical barrier to slow down the release of fuel and the diffusion of oxygen. The boron chemistry in the context of the physical constraints dictated by the natural material is analyzed. In solid wood and bamboo, the challenge is to use *in situ* mineralization and covalent grafting to overcome water solubility and leaching. In engineered wood composites and bio-based adhesives, boron moves from a passive additive to a structural element in the form of borate ester crosslinks. In flexible textiles, boron forms sol-gel architectures and synergistic combinations with phosphorus and nitrogen to achieve wash durability. Boron plays a crucial role in stabilizing high porosity nanocellulose aerogels and foams. The key challenges are identified to fulfil the potential of boron chemistry as a safe and sustainable approach for high performance natural materials.

## 1 Introduction

The global circular bioeconomy demands that timber, bamboo, natural fibers, and bio-based products are used to replace carbon intensive steel and concrete (Abed et al., 2022). However, the full potential of these renewable materials has not been realized, and these products are highly combustible. Upon heating, the plant cell wall polymers (cellulose, hemicellulose and lignin) undergo pyrolysis to release large quantities of levoglucosan, tars, etc., all of which are highly flammable (Bai et al., 2013; Senneca et al., 2020; Zhou et al., 2017). While delaying their ignition is important, the change of the thermal decomposition pathway away from oxidation and toward charring is the key for the fire retardancy of these bioproducts (Lowden and Hull, 2013; Sullivan and Ball, 2012). Among the wide range of flame retardant chemistries, boron-based compounds are particularly interesting because they have the potential to modify pyrolysis kinetics with low mammalian toxicity (Grams et al., 2024; Tatiya et al., 2020; Xu et al., 2022).

The fire retardancy mechanisms of boron compounds are distinct from that of gas phase radical scavenging as the majority of their reactions occur in the condensed phase (Popescu and Pfriem, 2020). Heating of boric acid and borates causes endothermic dehydration to form metabolic acid and boron oxide (Balcı et al., 2012; Huber et al., 2019). Physically, the low-melting point glassy layer creates an effective barrier preventing oxygen diffusion and heat transfer from the flame into the wood. Chemically boron acts as an acid catalyst for the decomposition of cellulose by catalyzing dehydration reactions to prevent levoglucosan formation (Hassan et al., 2020; He et al., 2023; Le et al., 2021). Thus, maximizing the formation of carbonaceous char is the key to limit the formation of flammable volatiles. As concerns grow over the persistence and toxicity of halogenated retardants, boron compounds are viewed as safer, smoke-suppressing alternatives. However, their safety profile is nuance, while they are less toxic than halogens. Specific borates face increasing regulatory scrutiny regarding reproductive toxicity under flameworks such as EU REACH. Furthermore, the different fungicidal and insecticidal properties of borates could lead to dual functions to resist both decay and thermal degradation (Marney and Russell, 2008; Stirling and Temiz, 2014).

Recent advancements focused on development methods to overcome the inherent water solubility of borates (Liu X. et al., 2022). This review presents an overview on the recent development of the boron-based fire retardants at different material hierarchies. The relationships between the material architecture and the retardant are discussed, starting from the bulk materials where the deep impregnation and *in situ* mineralization for the solid wood and bamboo. The flexible substrates where the wash durability is the key issue, and the sol-gel and covalent grafting on the textiles are reviewed. The fire retardancy of the engineered composites are discussed including the role of boron in bio-based adhesives and interfacial compatibility. With the use of porous materials, boron is no longer limited to be an additive, instead, it becomes the cross-linker in the fabrication of the nanocellulose aerogels/foams. By categorizing the recent progresses in different material physical forms, the boron chemistry is summarized for the adaption to satisfy the mechanical and thermal requirements of the various natural fiber applications.

## 2 Mechanisms of boron-based fire retardancy

Boron compounds interfere with the fire cycle on more than one physico-chemical fronts simultaneously. Most retardants act in either the gas phase or the condensed phase, in contract, boron species exert a multi-pronged mechanism at different temperature ranges. These versatilities give it the ability to target the relevant vulnerabilities of lignocellulose. The boron retardancy mechanisms can be categorized as: chemical catalytic action, physical barrier formation, dilution effects, and synergistic coupling effects with other heteroatoms.

**FIGURE 1:**
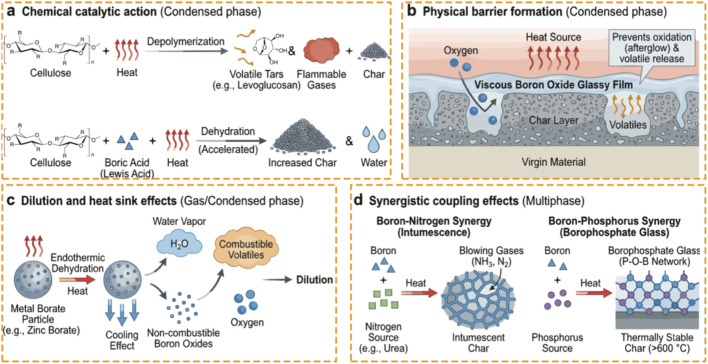
Mechanisms of boron-based fire retardancy. **(a)** Chemical catalysis acts as a Lewis acid to promote dehydration and char formation over depolymerization. **(b)** The formation of a viscous boron oxide glass creates a physical barrier that hinders oxygen diffusion and volatile release. **(c)** Endothermic decomposition provides heat sink and dilution effects through the release of water vapor. **(d)** Synergistic interactions with nitrogen and phosphorus enhance protection through intumescence and thermally stable borophosphate networks, respectively.

The most important fire retardancy action of boron in cellulosic substrates is the chemical catalytic modification of the pyrolysis reaction pathway (Wang et al., 2004; Yuan et al., 2025; Zhang L. et al., 2022). During the thermal decomposition, natural fibers undertake two competing reaction pathways: depolymerization that yields volatile tars (e.g., levoglucosan) and flammable gases, and dehydration that produces residual carbonaceous char and water (Jefferson et al., 2024; Sullivan and Ball, 2012). The flammability of natural fibers is determined by the dominance of the depolymerization. As shown in Figure 1a, boron compounds, especially boric acid and borates, act as Lewis acids that modify the reaction kinetics. During the heating, the acid releases protons or acidic species acting as a catalyst that lower the activation energy (E_a_) of the dehydrate reaction significantly. This kinetic modification shifts the thermodynamic competition, favoring low-temperature charring over the high-energy depolymerization pathway responsible for levoglucosan formation (Hall, 2005; Zhang et al., 2020).

Esterification of the hydroxyl groups at the C6 position of the glucopyranose ring, catalyzed by boric acid, blocks the sites that otherwise would have undergone intramolecular trans-glycosylation to form levoglucosan (Kawamoto et al., 2008; Wang et al., 2004). The carbon that would have been released in the volatiles as levoglucosan is thus prevented from exiting the solid phase. This results in an increased char yield and a decrease in effective heat of combustion of the volatiles that are released. It is often overlooked that boron treated materials usually decompose at lower temperatures than their untreated counterparts. This early degradation of the material is part of the char layer build-up that serves as a protective barrier from thermal flux that would otherwise occur at much higher temperatures (Wang et al., 2004).

As shown in Figure 1b, coupled with these chemical changes, a physical barrier is formed that, like the acid catalysis, is controlled by the phase changes that occur for the boron oxides with increasing temperature (Huber et al., 2020; Wang et al., 2004). As the temperature increases, boric acid dehydrates to form metaboric acid and then boron oxide (B2O3) (Balcı et al., 2012). At a temperature above 300 °C, these oxides form a viscous molten layer at the material surface (Servadei et al., 2021). This glassy film acts as a mass transfer barrier, reducing the diffusion of oxygen to the solid surface and the evolution of volatiles from the solid phase (Castanié et al., 2016; Uddin et al., 2017; Wang et al., 2004). The viscosity of the boron oxide film plays an important role in controlling the rheology of the film. On one hand, it has to be viscous enough to prevent the film from dripping away from the substrate at high temperatures. On the other hand, it has to be fluid enough to flow to seal any fissures that develop due to the thermal expansion of the solid (Castanié et al., 2016).

The glassy layer insulates the remaining virgin material from the heat and radiation sources, providing a physical barrier in addition to the chemical barrier created by the char (Yu et al., 2017a). The acid catalysis produces the char, while the boron glass encapsulates the char, preventing it from oxidizing (i.e., preventing afterglow) (Wang et al., 2004; Yu et al., 2017a). This is of relevance to natural fibers, which will often continue to smolder for hours after the ignition is stopped (Rein, 2009). The boron oxide film creates an airtight seal that prevents oxygen from reaching the carbon layer, preventing it from oxidizing. This is the primary reason why the continuation of fire for timbers and textiles treated with boron based FRs can be prevented at temperatures low for combustion, as this smoldering can lead to significant structural and aesthetic damage (Wang et al., 2004).

Thermodynamically, boron compounds contribute to fire retardancy through a dilution and heat sink effect mediated by endothermic decomposition and a release of non-combustible gases, as shown in Figure 1c. This effect is especially prevalent with metal borates such as zinc borate due to their high water of hydration, the dehydration of which occurs over a broad temperature range (290 °C–415 °C). This coincidentally encompasses the decomposition window of many natural polymers and bio-composites (Huber et al., 2019; Wang et al., 2004). The vaporization of this water of hydration requires high thermal energy, resulting in a cooling effect. Additionally, the release of water vapor, as well as inert boron oxides, into the gas phase dilute the concentrations of combustible volatiles and oxygen, driving the mixture below its lower flammability limit. Although it has less dominant effect than that from the condensed phase charring, dilution represents a critical complementary process to delay time-to-ignition and reduce peak heat release rate.

The complexity of boron fire retardancy is much increased when combined with other elements in the synergistic systems, most notably nitrogen and phosphorus. These interactions yield more than an additive effect, but rather a multiplicative one, in terms of performance efficiency (Re et al., 2020). In the boron–nitrogen systems, commonly for the textile finishing, the presence of nitrogen sources, such as urea or melamine facilitate the formation of voluminous, swollen char structures known as intumescence (He et al., 2022; Li Y. et al., 2024; Xie et al., 2013). Nitrogen compounds generate non-combustible gases (ammonia, nitrogen) that expand the char in the same way as blowing agents expand a polymeric foam, with the boron stabilizing the cell walls of the resultant structure (Doğan et al., 2010; Li Y. et al., 2024; Ma et al., 2024).

Similarly, as shown in Figure 1d, phosphorus and boron act synergistically through the formation of borophosphate glasses (Koudelka and Mosner, 2000; Qiu et al., 2008; Re et al., 2020; Sharmin et al., 2013). While phosphorus is a powerful acid source to promote lower temperature charring, the resulting carbonaceous structure is brittle and susceptible to oxidation under the highest temperature fire exposure (Li Y. et al., 2024; Schartel, 2010). The incorporation of boron to stabilize the phosphorus rich residue increases the thermal stability of the char above 600 °C (Doğan et al., 2010; Re et al., 2020; Sharmin et al., 2013). This Phosphorus–Boron (P-B) interaction is important for achieve high-performance where a structurally sound char must be retained under fire exposure. The phosphorus–oxygen–boron (P–O–B) bond created in a cross-linked network is more thermally, oxidatively, and hydrolytically stable than phosphate or borate networks alone (Koudelka and Mosner, 2000; Qiu et al., 2008; Sharmin et al., 2013). In these systems, the B/P molar ratio is a critical parameter since it regulates the glass transition temperature (T_g_) and viscosity of the melt, ensuring the barrier remains intact without dripping. Furthermore, boron contributes a distinct “anti-glow” mechanism by stabilizing the phosphorus-rich char against oxidation at high temperatures, preventing the flameless consumption of the carbon skeleton.

These mechanisms including the catalytic promotion of char, the glassy barrier and endothermic cooling, the synergistic network stabilization, provide the necessary background to understand the materials and their applications. Regardless of how the boron compound is applied, deep impregnation into timber, covalent grafting onto nanofibrils, the underlying goal is to suppress the volatile generation and preserve the carbon backbone through these orchestrated chemical and physical processes. The following sections will explore how these mechanisms are applied to distinct material hierarchy, beginning with the bulky structure of solid wood and bamboo.

## 3 Solid wood and bamboo modification

Solid wood and bamboo have an anisotropic hierarchically porous structure and are not easily fire-retardant treated compared to fibrous and particulate lignocellulosic materials. The vascular systems (i.e., tracheid in softwoods, vessel in hardwoods, and vascular bundles in bamboo) form continuous pipelines that exacerbate the chimney effect and boost flame spread in the matrix. Generally, boron salts are on the wood surface to improve the fire resistance, which are prone to hygroscopicity, poor penetration, and leaching. Consequently, the fire retardancy will be significantly weakened once the charred surface is mechanically destroyed. For the traditional treatment, flame retardants are simply filled into the porous lignocellulosic matrix. Recent fire-retardant treatment approaches are aiming to actively engineer the lignocellulosic structure with fire-retardant wooden materials. The hierarchical porous lignocellulosic structure can be served as the template for mineralization, chemical grafting, and densification process.

### 3.1 *In-situ* mineralization and leaching resistance

**FIGURE 2:**
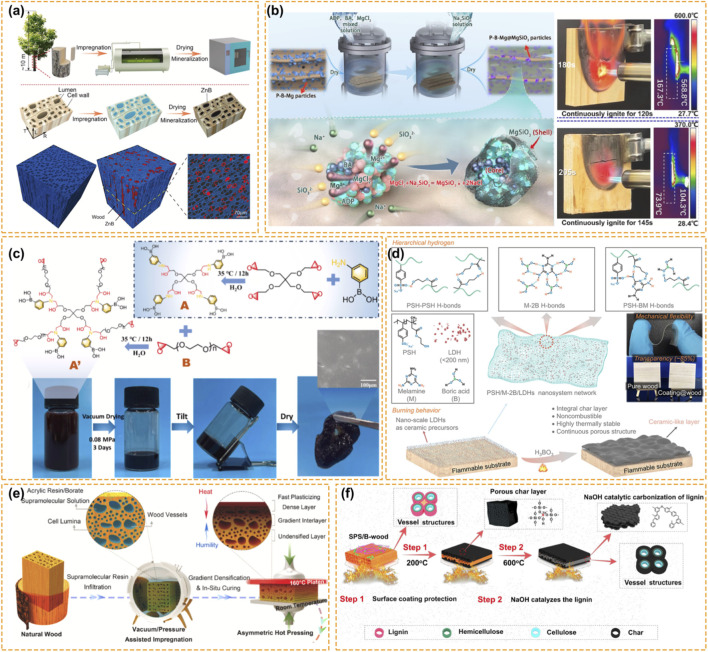
Boron-Based Fire Retardancy for Solid Wood and Bamboo. **(a)** Schematic for the fabrication of mineralized wood and 3D rendering of a small region-of-interest from the analyzed X-ray tomogram (Zhou H. et al., 2023). Reproduced with permission. Copyright 2023, Elsevier. **(b)** Schematic of the *in situ* synthesis of the P–B–Mg@MgSiO 3hybrid flame retardant with a core-shell structure in wood and infrared thermal imaging system for flame retardancy assessment (Zhang A. et al., 2024). Reproduced with permission. Copyright 2024, Elsevier. **(c)** The reaction mechanism of PTGE and PBA to synthesis prepolymer A, and the *in situ* synthesis of HPB-PBA (polymer A′) via polymer A and PEGDE (polymer B) in bamboo (Zhang Y. et al., 2024). Reproduced with permission. Copyright 2024, Elsevier. **(d)** Schematic diagram of the hierarchical interactions between PSH chains and the supramolecular molecules in PSH/BM/LDH nanosystem network (Guo et al., 2025). Reproduced with permission. Copyright 2025, Springer Nature. **(e)** Schematic demonstrating the top-down approach of gradient densification assisted with aqueous acrylate/borate supramolecular resin impregnation to transforming bulk natural wood directly into multifunctional unilateral gradient densified wood (Fan et al., 2024). Reproduced with permission. Copyright 2024, Elsevier. **(f)** Schematic illustration for flame retardant mechanism of SPS/B-wood sample (Wang et al., 2021). Reproduced with permission. Copyright 2021, Springer Nature.

Instead of allowing the empty space to act as an oxygen transporter, the void volume is considered as a container to be filled with inorganic flame retardants. Such *in situ* mineralization is achieved by mimicking the biomineralization process, in which insoluble fire-retardant particles are incorporated deep into the cell’s lumen. As shown in Figure 2a, Zhou et al. synthesized nanosized zinc borate (ZnB) into the hierarchical porous structure of wood. Instead of covering the surface with a continuous shield that prevent the contact between the oxygen and the flammable gases and the subsequent combustion, ZnB particles are latent precursors, which melt to form a uniform and non-combustible film upon burning (Zhou H. et al., 2023). Such a molten film acts as a thermal insulator and a crosslinking agent for the char that forms a 3D skeleton keeping the original dimensions even when the organic matrix has completely vanished. The LOI (Limiting Oxygen Index) is the minimum oxygen concentration needed to sustain combustion of a material under test conditions) significantly increase to 41.2% while the peak of the heat release rate drops by almost half (Zhou H. et al., 2023).

One major drawback of boron lies in its water solubility, which leads to a rapid leaching in outdoor applications. For this reason, some researchers used a strategy, in which soluble precursors were introduced into the wood structure and subsequently transformed *in situ* into an insoluble compound. As shown in Figure 2b, Zhang et al. developed a core-shell flame retardant (P-B-Mg@MgSiO3) composed of a soluble phosphorus-boron-magnesium core covered with an insoluble shell formed *in situ* by magnesium silicate (Zhang A. et al., 2024). The core-shell approach overcomes the problem of water solubility without reducing the performance of boron. The shell prevents leaching during the service life of wood. When the wood is burned, the shell broke due to the thermal expansion, releasing the active compound, boron. Following the same strategy, melamine formaldehyde (MF) resin can also be used to fix the boron within the wood porous structure. Boron species were doped into MF resin microspheres polymerized into the wood cells, so that the leaching resistance was significantly improved while keeping the thermal stability offered by boron (Lin et al., 2021). In this approach, inorganic borates need to be hybridized with hydrophobic polymers which trap the compound inside the cells to overcome leaching.

### 3.2 Chemical grafting and gelation networks

Besides the physical filling of the porous structure of the wood, attempt have been made to chemically modify the cell wall, resulting in treated wood products that are much more durable with more effective fire retardancy. The cell walls are rich in hydroxyl groups, either through cellulose or lignin, that can be covalently modified to become a part of the macromolecular structure, particularly for bamboo which has no transverse rays. Impregnation of such structure is particularly difficult because of the poor transport within bamboo. Zhang et al. used tannic acid and boric acid to construct a borate-catechol covalent gel network within the xylem of bamboo (Zhang et al., 2023). The borate-catechol gel network not only fills the xylem and the bamboo pores but also alleviate swelling/shrinking of the bamboo through mobility of the gel, which significantly improved its durability and mold resistance.

Further developments have been presented employing hyperbranched polymers developed specifically for the interaction with biomaterials. As shown in Figure 2c, hyperbranched polyethers with phenylboronic acid groups were synthesized and high retention of the boron (60.6%) was observed in bamboo through covalent bonding with the bamboo cell wall (Zhang Y. et al., 2024). The hyperbranched polymer fills and swell the bamboo cell walls to replace hygroscopic water with flame retarding polymer. Besides lowering flammability, an improvement in dimensional stability was also observed. Another development was the chemical modification of Scots pine with covalent attachment of boron-functionalized benzoates to the cell wall (Ehrhardt et al., 2021). Covalent bond of boron-functionalized benzoates significantly lowers the mass loss, indicating an earlier catalytic onset of char formation that protects the bulk material. It was also discovered that lignin can activate room temperature phosphorescence of borax through non-covalent interactions that enhance the spin-orbit coupling, which gives rise to phosphorescence (Zhai et al., 2025). The work also showed the intimate interaction between boron and the aromatic structures of lignin. Besides covalent bond, chelation may also be used to increase the amount of boron loading into the natural material. In the impregnation of traditional boric acid solution, self-condensation or self-esterification may occur resulting in a number of polyborates, which crystallize out inside the lumen of wood rather than attach to the cell wall (Yamauchi et al., 2007). By using a chelating ligant to form a stable organoboron complex, the electrophilicity of the boron center is modified. This change suppresses the self-polymerization of the boron species allowing the molecular complex to diffuse into the amorphous regions of the natural polymer matrix prior to exchanging the chelating ligands for stable ester links to the cellulosic hydroxyls (Miyaz et al., 2003; Yamauchi et al., 2007). This approach dramatically enhances the boron incorporation compared to impregnation from the salt alone. Since the boron is present as molecular cross-linker instead of just a filler, the matrix is rigidified, and the mechanical properties of the treated product are improved. Thermally, because the boron is present in the molecular level, a lower threshold for catalytic dehydration is needed leading to a much more homogeneous char formation (Wang et al., 2004).

### 3.3 Densification and structural synergies

The denser the wood, the more energy (fuel) it provides. Also, the denser wood tends to have a greater thermal inertia and thus it is more difficult to be ignited. Recent studies have combined mechanical densification with boron treatment. Densified wood is processed by hot-pressing the wood of that the lignin is partially removed, to form a compact wood with outstanding strength. Boron chemistry can be combined into the process so that the products can be fire resistant. Li et al. demonstrated a non-adhesive laminated wood where the borate ions cross-linked the remaining hydroxyl groups in the delignified fibers (Li et al., 2025). In this case, boron serves as both fire retardant and binder that the flammable synthetic glue is no longer needed. The laminated wood obtained a V-0 rating (higher fire safety) with an improved mechanical property.

Inspired by nature, as shown in Figure 2e, Fan et al. proposed a gradient densification and *in situ* curing of polyacrylic acid/borate resins for wood (Fan et al., 2024). By tailoring the moisture-thermal fields during hot-pressing, a densified wood with a density gradient along its thickness was developed. The surface of the densified wood is harder than its core, exhibiting an optimal balance between hardness and fire resistance with minimized density. The density gradient effectively suppressed the smoke release by 74%. This result indicates that how the retardant is delivered to wood is as important as the chemical composition of the retardant. Combination of compression and impregnation process benefits the low-grade timber such as fast-growing poplar. Resin impregnation together with transverse compression can significantly improve their fire resistance (Cheng et al., 2022).

### 3.4 Functional surface barriers and ceramization

For some wood species, particularly those with heritage structures, deep impregnation may be impractical. In such cases, surface fire retardant coating to achieve a ceramizable skin can be used. This coating polymer is usually cured upon heating to form a hard shell of ceramic or glass. As shown in Figure 2f, Wang et al. proposed a coating made of sodium polysilicate and boric acid. The two compounds will react during the combustion to form Si-O-B and Si-O-Si structures (Wang et al., 2021). Such bridges isolate the heat and smoke transfer. The addition of sodium hydroxide to this kind of formulation favors the formation of high-quality char from the underlaying lignin.

Often, transparency is required for wood coatings to preserve its grain aesthetic value, as shown in Figure 2d, Guo et al. reported a transparent fire-proof coating based on layered double hydroxide (LDH) nanosheets and supramolecular melamine di-borate (Guo et al., 2025). Upon heating, a porous vitreous protective layer is formed reducing the total heat release by almost 80%. Hybridized networks of graphene oxide (GO) and boric acid can also act as fire-warning sensors (Che et al., 2024). In fact, the GO/BA layer provides a rapid and sensitive alarm response, simultaneously enhances the charring effect, raising the LOI values to >75%. Such convergence of sensor function and passive protection can be envisaged as one of the most promising future developments of boron-based functional surfaces.

Summing up, modification of solid wood and bamboo has evolved from simple salt impregnation to complex molecular and structural engineering. Whether the lumens are mineralized, cell walls are covalently grafted or ceramizable skins are created on the surface, the common denominator is the use of boron to change the thermal degradation direction toward char and glass formation, reinforcing the lignocellulose scaffold against fire.

## 4 Engineered composites and bio-based adhesives

The transition from solid timber into engineered wood products introduces the potential to consider fire retardancy strategies. Whereas solid timber, for all practical purposes, has the microstructure locked in by nature, the ability to remake the wood/adhesive matrix from constituent fibers (Wood Plastic Composites/WPC, Oriented Strand Board/OSB, plywood etc.) enables the microstructure and chemical nature of the interfaces to be controlled and modified to suit the application. It is in this field that boron can perform a dual function, not only acting as a fire suppressant but also modifying the chemical interface and the rheology, curing rate and cross-linking density of the matrix. The challenge here is to not only introduce a high level of inorganic retardant but to do so without destroying the mechanical properties of the wood composite material and the adhesion of hydrophilic fibers to hydrophobic matrices.

### 4.1 Interfacial dynamics in thermoplastic composites

Flammability of wood plastic composites (WPCs) and natural fiber-reinforced polymers is increased by the presence of a highly flammable thermoplastic matrix, such as polyethylene (PE), polypropylene (PP), or polylactic acid (PLA). The incorporation of boron species in a multiphase system is usually trade-off between the promotional charring effect and the mechanical embrittlement. Zinc borate (ZB) has been demonstrated to work as a char promoter more effectively than tin-based alternatives in polyvinyl chloride (PVC) wood flour composites, as it acts both in bulk and surface (Liu J. et al., 2022). Water of hydration is released at early stage of combustion, which promotes dilution of the combustible gas mixture and formation of a porous ceramic residue that protects the underlying polymer.

Boron is particularly efficient when hybridized with other inorganic particles to influence the rate of burning in WPCs. A significant burning rate suppression (around 80%) has been achieved with the addition of ulexite (sodium calcium borate) to flax/PLA composites (Avci et al., 2022). However, the significant thermodynamic enhancement is compromised with a tensile property reduction by as much as 20%, due to a poor stress transfer at the filler-matrix interface (Avci et al., 2022). To overcome this drawback, a synergistic approach with silicon dioxide (SiO2) was recently used together with zinc borate and was found that the burning rate was reduced by 36.6% in jute/epoxy hybrids, while the impact strength of the composites was enhanced by 2.5 times (Canıtez et al., 2023). The rigid inorganic particles at suitable ratio can act as crack arresting sites providing the dispersion effectively to prevent agglomeration.

The structural complexity of WPCs may conceive more complicated approaches to resemble sandwich structures. Layering the wood flour/high density PE (HDPE) core with polycarbonate (PC) doped with boric acid offers the chance to use PC as a nucleating agent for crystallization and a rapid-charring skin (Zhang et al., 2021a; Zhang J. et al., 2022). The boric acid treatment of wood flour enhance the char residue by more than 100% compared to that of the pure polymer, enabling a modification of the degradation route of the wooden component, so as to support the carbonaceous skeleton of the sandwich (Zhang et al., 2021b). This enables an effective decoupling of the resistance to fire of the surface and mechanical strength of the core. The hybrid use of ammonium polyphosphate (APP) with boric acid-borax precursors with bamboo-HDPE system showed a remarkable synergetic effect, in which phosphorus acts as a carbonization source and boron species stabilize the resulting char, enabling to achieve the maximum LOI values through a combination of the barrier and char promotion system (Kumar and Chauhan, 2022).

### 4.2 Flame-retardant boron-based adhesives

**FIGURE 3:**
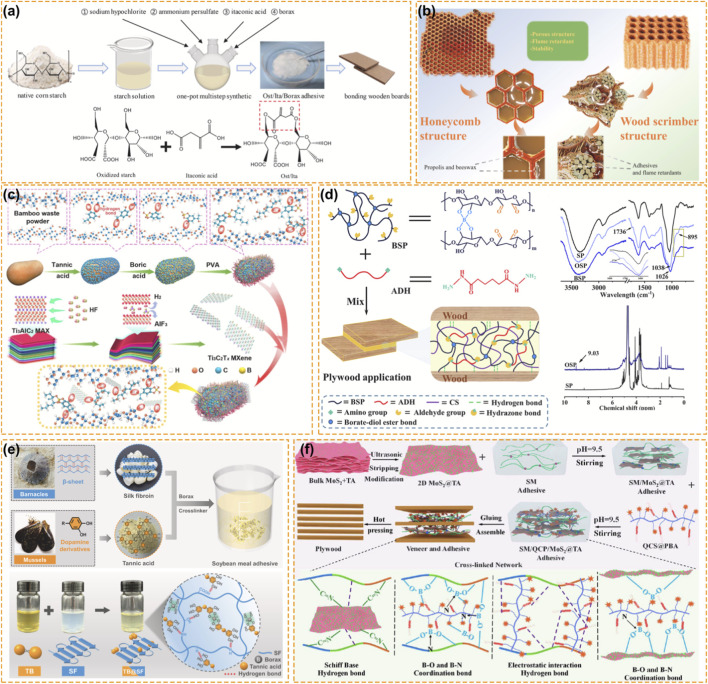
Boron-Based Fire Retardancy for Engineered Composites and Bio-Based Adhesives. **(a)** Preparation of the Ost/Ita/Borax adhesive and the reaction formulae showing the synthesis of the Ost/Ita/Borax adhesive (Chen Y. et al., 2024). Reproduced with permission. Copyright 2024, Elsevier. **(b)** Source of inspiration and preparation process of the flame-retardant wood scrimber samples (Song et al., 2025). Reproduced with permission. Copyright 2025, Elsevier. **(c)** Design strategy for multifunctional bamboo waste composite (Wang et al., 2024). Reproduced with permission. Copyright 2024, Elsevier. **(d)** The schematic diagram of the synthesis principle of BSPC-ADH adhesive and FTIR spectra (Zhou et al., 2022). Reproduced with permission. Copyright 2022, Elsevier. **(e)** Design strategies for soybean meal adhesives inspired by barnacles and mussels and chemical structure of TB@SF hybrids (Jiang et al., 2023). Reproduced with permission. Copyright 2023, Elsevier. **(f)** flowchart of adhesive and plywood preparation and reaction mechanism of SM/QCP/MoS2@TA adhesive (Zeng et al., 2024). Reproduced with permission. Copyright 2024, American Chemical Society.

The most groundbreaking development in recent years is the transition of boron from an additive to a major cross-linking agent in the development of flame-retardant bio-adhesives as shown in Figures 3 a–f. Due to their poor water resistance and low bonding strength, soy protein and starch adhesives are unable to entirely replace formaldehyde-based resins. Boron chemistry and the formation of borate ester bonds with hydroxyl and vicinal diol groups not only improve the water resistant, but also allow the adhesive to be flame retardant.

For starch-based adhesives, as shown in Figure 3a, the addition of borax allows itaconic acid to form ester bonds with starch and starch chains to form ester bonds with the borate. A dense skeleton is thus formed, in which the adhesive has a LOI value of 30.9% (Chen Y. et al., 2024). The same chemistry can also be used for soy protein isolates where dynamic covalent bonds can be formed with the addition of sodium tetraborate. These reversible covalent bonds can dissipate the energy by sacrificial bonding inspired by the cutin structure of plant cell walls, which allows the adhesive to have excellent flame retardancy, toughness and weatherability (Huang et al., 2024).

Bio-mimicking boron chemistry can be applied to fabricate complex nanohybrids where soybean meal is combined with molybdenum disulfide (MoS2) and phenylboronic acid. Researchers successfully replicated the microstructure of a dragonfly wings where boron-nitrogen coordination bonds were exploited to maximize interfacial interactions (Zeng et al., 2024) (Figure 3f). Through this technology, adhesives with superior fracture toughness and mildew resistance can be obtained. Furthermore, hyperbranched phenylboronic acid siloxane nanoclusters enable a dual cross-linking strategy of soy matrices (Shao et al., 2024). The boron is not only the cross-linker to improve the wet shear strength (1.12 MPa), but also a char promoter and a fungicide that can promote the formation of char hindering the fungi attack.

Combining boron and naturally derived polyphenols such as tannic acid will have a further synergistic effect. For soy-silk fibroin adhesives, as shown in Figure 3e, the addition of borax and tannic acid can form a robust skeleton, which can protect the protein from hydrolysis and thermal oxidation with a LOI value of 30.1% (Jiang et al., 2023). For a simple system where borax was combined with core-shell nanohybrids, the formation of hard core and flexible shell structure within the glue line was effective for particleboard to achieve V-1 fire rating without compromising the elasticity required in furniture applications (Zeng et al., 2022).

### 4.3 Glued engineered panels

Glued engineered wood panels, such as plywood, oriented strandboard (OSB), and laminated bamboo may require fire retardant treatment. Their fire performance is dependent on the flammability of both the lignocellulosic component and the adhesive layer. The glue line can be a weak point in fire, as a continuous path for flame spread. Laminated bamboo treated with a combination of melamine urea formaldehyde (MUF) and boron-based retardants provides a powerful nitrogen/boron synergy. The melamine component acts as a blowing agent and boron stabilizes the foam, producing an intumescent effect at the bond line, which reduces the heat release and smoke production (He et al., 2024). Plywood treated with a multicomponent retardant mixture, including guanidine and disodium octaborate, reduces the pyrolysis temperature of cellulose, promoting dehydration and carbonization in a way that the total heat release can be reduced by almost 83% (Yu et al., 2021). In wood scrimber, which is made from wood screams crushed at high temperatures to a high density beam, impregnation with a mixture of sodium silicate and sodium tetraborate allows the mixture to be contained in the voids within the crushed wood. Upon heating, as shown in Figure 3b, the mixture releases its crystalline water and forms a silicon/boron glass, which blocks the paths of smoke release, reducing the total smoke release by over 43% (Song et al., 2025). Even honeycomb panels manufactured from a mixture of pine bark and cones bonded with a urea formaldehyde (UF) modified with boric acid are non-flammable (Efe, 2022).

However, the use of boron in engineered panels may be a trade-off. Although boron improves the fire performance of the products, in many cases, the presence of the inorganic salt affects the adhesive curing. High concentrations of boric acid can reduce the shear strength of wood-polyurethane bonds, due to the influence of boric acid on the wettability and chemical compatibility of the wood-polyurethane interface (Ciglian and Reinprecht, 2022). Conversely, the production of OSB coated with a polyelectrolyte complex of sodium polyborate and polyethylenimine may avoid such problem. The coating adds only 5.8% of mass to the OSB, while it confers self-extinguishing behavior via an intumescent mechanism that preserves the mechanical properties of the panel (Rodriguez-Melendez et al., 2024).

The future of fire retardant engineered wood composites will be dependent on the chemical integration of boron through the formation of borate ester cross-links in bio-based adhesives, or through the synergistic stabilization of intumescent chars in laminates, or as the boron component of some multicomponent fire retardant. Due to its unique chemical versatility, boron allows the production of sustainable materials with high-end performance.

## 5 Textile and flexible fiber finishing

Deeply impregnated timber with flame retardant chemical solutions may achieve relatively high mass loadings, with no adverse effect. Textile coated with flame retardant is highly constrained by the added weight to the fabric, the fabric hand, and the wash durability. Simple borax and boric acid impregnations have been widely used to treat cotton and jute for centuries. These treatments perform well in terms of the initial flame retardation imparted to the textile. However, because of the high solubility of the inorganic borates in water, they do not meet the current laundering standards. Consequently, growing research has been seen on new approaches to anchor boron moieties covalently to the cellulosic backbone or to encapsulate the boron in hybrid organic–inorganic networks. Instead of applying boron as a simple deposition onto the fiber, boron is frequently being grafted to the cellulosic backbone and applied in conjunction with other heteroatoms.

### 5.1 Synergistic phosphorus-nitrogen-boron (P-N-B) architectures

An introduction of a termolecular P–N–B system was presented, with all three playing their part in the fire retardancy: the acid source, the blowing agent, and the glass former. When the P–N–B system is implemented in the presence of an oxidizable substrate like cotton, it will be phosphorus that ensures dehydration beginning at low temperatures; nitrogen will generate a series of non-combustible gases, whereas boron will finally fix the phosphorus in the intumescent structure, with the formation of borophosphate glasses.

Recently, the strategy of making a P–N–B mixture was substituted by the synthesis of single P–N–B molecules. The synthesis of a so-called H-shaped flame retardant, with a 2:3:1 P–N–B atomic ratio (Jin et al., 2024), shows profound duality. The molecule not only covers the cellulosic fiber, but also, at the same time, forms at least 3 covalent bonds with the cellulose chains. The deterioration of the textile material due to the strong acidic environment, usually accompanying P–N-based FR treatments, is compensated. Consequently, the LOI of the cotton treated with this system reaches >41%. It was also reported that the tensile strength of the treated fiber was improved by more than 20% (Jin et al., 2024).

Similar structural strengthening effects were observed in spiro-cyclic compounds. By creating the diboraspiro rings with phosphorus and nitrogen, a highly efficient retarding effect is obtained at relatively low weight gains (Lan et al., 2025). The spiro structure imparts rigidity to the molecule, whereas the P–N–B combination ensures the realization of the solid-gas phase, releasing ammonia and water vapor that dilutes the combustion zone and the cross-linked borophosphate char in the condensed phase. This synergistic stabilization is critical. Without boron, phosphorus-based chars are often brittle and prone to oxidative degradation at high temperatures. The inclusion of boron, even in a small molar fraction, produces a ceramic character in the residues of heat-treated fabrics; consequently, at 35 kW/m2 heat flux, the cotton fabrics’ warp and weft structure are maintained (Zhang Z. et al., 2024; Chen et al., 2024b).

### 5.2 Sol-gel architectures and inorganic hybrids

Although the issue of solubility is addressed using a pure organic synthesis, there is growing interest to develop a sol-gel process, which could produce inorganic networks *in situ* on the fiber surface. In the binary oxide system, the combination of boron into the silica (SiO2) sol resulted in an interesting thermal behavior. While the silica provides a structural skeleton which has high melting temperature, the boron functions as a low melting point phase material. The boron phase melts upon heating and seals the silica gel porosity creating a hermetic seal (Zhou C. et al., 2023). When the combination of silica and boron is used as a protection system, the silica prevents the low viscosity boron melt to drip and conversely the boron seals the cracks produced by the thermal expansion of the rigid silica shell (Li G. et al., 2022).

Coupling agents and metal salts were also shown effective to enhance the efficiency of hybrid systems. The hybridization of silica sols with ZB and silane coupling agents was found to reduce the peak of the heat release rate considerably with the assistance of a glassy barrier formation as well as an endothermic dehydration (Li D. et al., 2022). In such a system, the use of silanes such as 3-glycidoxypropyltrimethoxysilane, is crucial as the silica sol could form a continuous layer instead of isolated clusters, which is very important to the fabric handle. Ionic liquids containing boron were also synthesized by the sol-gel process, which produced an effective finish that did not lead to flame spread and conserved the cotton tensile strength (El Messoudi et al., 2022).

### 5.3 Challenges of durability and covalent fixation

One of the drawbacks of boron-based textile finish is their wash durability. Current research considers chelation or ion-transfer that traps boron into insoluble complexes. The synthesis of chelated boric acid complexes with amino-trimethylene phosphonic ligands lead to a finish withstanding 50 washing cycles with a LOI around 30% (Zhu et al., 2023; Zhu W. et al., 2022). The underlying physics involves the formation of stable coordination bonds that resist hydrolytic cleavage during washing but readily dissociate to release active boron under thermal stress/fire to achieve flame retardancy.

**FIGURE 4:**
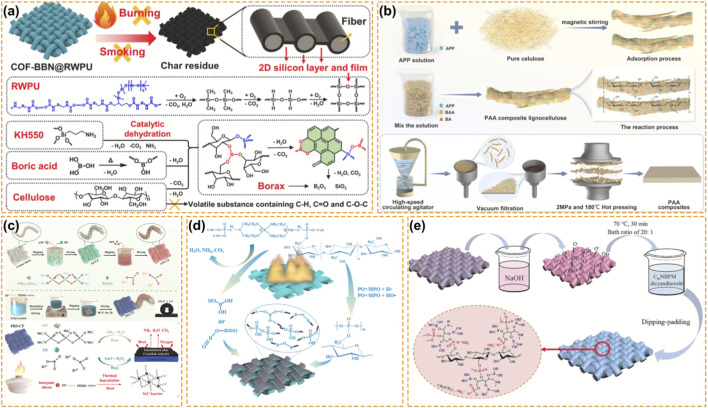
Boron-Based Fire Retardancy for Textile and Flexible Fiber Finishing. **(a)** The FR mechanism of cotton fabrics with a polydimethylsiloxane-based polyurethane (Chen et al., 2024a). Reproduced with permission. Copyright 2024, Elsevier. **(b)** Schematics of the preparation process of aqueous flame retardant composites (Yin et al., 2025). Reproduced with permission. Copyright 2025, Elsevier. **(c)** Schematic illustration of preparation and flame-retardant mechanism of flame-retardant superhydrophobic cotton fabric (Su et al., 2023). Reproduced with permission. Copyright 2023, Elsevier. **(d)** Flame retardant mechanism of diethanolamine borate methylene phosphonium ammonium salt (Zhang Z. et al., 2024). Reproduced with permission. Copyright 2024, Springer Nature. **(e)** Schematic diagram of fabrication of CmNBPM cotton fabric (Shi et al., 2023). Reproduced with permission. Copyright 2023, Elsevier.

Another way to deal with the durability issue is the hydrophobisation of the textile. Coupling the boron-based formulations with hydrophobic polymers such as polydimethylsiloxane (PDMS), lead to a so-called water-repellent fire armor. In these systems, phase separation between the boron-based flame retardant and the hydrophobic coating PDMS lead to a micro-nano rough structure (Chen et al., 2024c). This structure, as shown in Figure 4a, allows to obtain a superhydrophobic fabric (contact angle >140°) while prevents the solubilization of the water-soluble boron-based flame retardants by isolating them from water during laundering (Chen et al., 2024a). Such systems allow to obtain a fire-retardant fabric avoiding the contact of the flame retardant with water. The self-extinguishing behavior is preserved after rigorous washing cycles.

### 5.4 Substrate specificity: natural fibers and blends

For other lignocellulosic fiber like jute, applying borax could significantly extend the time to flame spread, but might cause the breaking load of the fabric to drop to some extent (Repon et al., 2021). For those fibers containing a relatively low degree of polymerization, the conflict between fire safety and mechanical property is more difficult to resolve. For protein fibers such as wool, the chemistry should be adjusted according to the presence of amine and amide groups. Functionalizing caramel with borate groups via Schiff base reactions to retard wool by promoting the charring process through a condensed-phase mechanism of protein backbone was reported (Cheng et al., 2021).

In Figures 4b–e, boron always plays a critical role in some synthetic-natural blends like nylon/cotton or polyester/cotton, in which the thermal degradation behaviors of the two components are quite different. For instance, in nylon/cotton blend, boron compound produce a ceramic-like char to avoid the “scaffolding effect” where the cotton char could support the melt nylon which always results in the severe burning of the fabric (Liu J. et al., 2022). Similarly, in polyester/cotton blend, P-N-B coating could greatly change the thermal degradation behaviors by suppressing the melt-dripping of polyester and promoting the charring of cotton (He et al., 2022; Li Y. et al., 2023). As a result, the boron has been considered as one of the most promising elements for textile finishing.

To summarize, with the development of the textile industry, the boron compound as a classical fire retardant has undergone the transformation from simple impregnation of salts to precise construction of molecular and supramolecular architectures. The synergic reinforcing effect of “H-shaped” molecule, the dynamic healing effect of the binary sol-gel coating, the hydrophobic protection effect of durable finishing, etc., all these cases show that the boron chemistry can be adapted to the rigorous demands of flexible, washable, and durable textiles.

## 6 Aerogels, foams, and hydrogels

**FIGURE 5:**
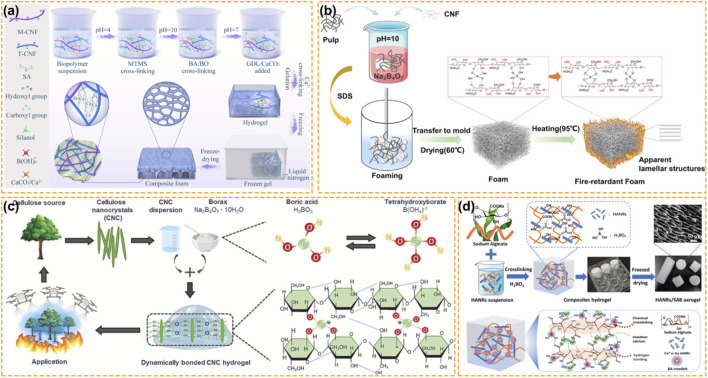
Boron-Based Fire Retardancy for Aerogels, Foams, and Hydrogels. **(a)** Schematic illustration of the cellulose-based composite foam fabrication process (Jiang et al., 2021). Reproduced with permission. Copyright 2021, Elsevier. **(b)** The fabrication route for laminar cellulose-based foam (Hou et al., 2023). Reproduced with permission. Copyright 2022, Royal Society of Chemistry. **(c)** Schematics of the flame-retardant hydrogel synthesis and its proposed application for the prevention of fire in cellulosic materials (Koparipek-Arslan et al., 2024). Reproduced with permission. Copyright 2024, Elsevier. **(d)** Preparation process and mechanism of the binding mode of the HANRs/SAB composite aerogel (Zhu J. et al., 2022). Reproduced with permission. Copyright 2022, American Chemical Society.

Boron chemistry approaches the highest level of complexity in the development of advanced porous materials. While timber, textiles or insulation board are materials, in which the boron compound is added to improve their fire performance, in aerogels, foams or hydrogels, boron compound itself acts as building blocks to the material (Figures 5a,b). Rather than a fire retardant, boron compounds are often used as essential cross-linking agents, which regulate the rheology, pore structure and mechanical properties of the 3D materials. This is a lightweight thermal insulation, where boron compound acts as both fire retardant and building blocks for the materials structure.

### 6.1 Boron-crosslinked hydrogels as active heat sinks

Borax and boric acid react with the vicinal diols on cellulose, alginate, and poly (vinyl alcohol) (PVA) to form dynamic covalent bonds. With such reaction, borax and boric acid can go from liquid to viscous solid without the need for toxic synthetic cross-linkers. As shown in Figure 5c, Koparipek-Arslan et al. used cellulose nanocrystals (CNCs) extracted from wood to be cross-linked by boronate ester bonds (Koparipek-Arslan et al., 2024). As a hydrogel, the material transitions from a liquid to a soft-solid, with tunable elasticity. Such network is dynamic, and the reversibility of the boronate bond allows the hydrogel to be self-healing. Applied as a coating, this hydrated network can delay ignition by 30 s not only by the heat retained by the bound water, but also by this network to form a coherent, non-flammable skin, which can re-heal if it is broken prior to carbonization (Koparipek-Arslan et al., 2024).

This ability to cross-link is critical for super-absorbent hydrogels where the end goal is to retain the water as much as possible–a property that is also useful for fire safety. Tanpichai et al. prepared hydrogels from cellulose where the borax cross-linking improves the swelling ratio to about 900% (Tanpichai et al., 2022). In a fire, these hydrogels act as active heat sinks. The boron network locks the water into the polymer structure. In the presence of a flame, the controlled release of water creates a steam barrier that dilutes the oxygen and cools the substrate. At the same time, the borax promotes immediate charring on the surface, which slows down the flame spread.

### 6.2 High-performance aerogels: the porosity paradox

Bio-aerogel has an ultra-high porosity (i.e., >95%) and high specific surface area so that it is highly combustible. Boron based coating can be applied to the skeleton of nanofibrils with a glassy barrier precursor. Wang et al. used bacterial cellulose aerogels (BC) with synthesized zinc borate (ZnB) particles (Wang et al., 2023). It was found that adding ZnB did not collapse the fragile porous structure, rather, it reinforced the fiber bundles. On heating, ZnB dehydrates and releases oxides (ZnO and B2O3) to insulate the flammable fibrils. This successfully neutralizes the convective heat transfer that normally plagues low density aerogels, with a resultant heat release capacity of only 8 J/g·K, which is considered as a very low number for an organic aerogel (Wang et al., 2023).

To increase the mechanical stability of the fragile networks, a double cross-linking method can be adopted. Yue et al. used a cellulose nanofiber/sodium alginate aerogel based on the “egg-box” structure of Ca2+ and borate cross-linking (Yue et al., 2023). Calcium ions reinforce the alginate chains through the so-called egg-box structure. The interpenetrating network formed by the boric acid not only rigidifies the network, but also compacts the carbonized layers formed upon combustion. As a result, the condensed and gaseous phases work together to form a V-0 material with a LOI of 35.6%. Also, hydroxyapatite nanorods (HANRs) containing alginate/boric acid aerogels form a bone-mimetic structure by cross-linking alginate with boric acid to form a solid matrix and reinforcement with thermally stable HANRs, as shown in Figure 5d. Based on the concept, alginate/boric acid/hydroxyapatite composites with a smoke release of 11.3 m^2^/m2 has been prepared (Zhu J. et al., 2022). HANRs prevent oxidation of organic matrices and act as a thermal barrier.

### 6.3 Synergies in phosphorylated and hybrid systems

Molecular-level interactions of the porous materials may tailor through phosphorylation. Zhou et al. prepared the aerogel based on the phosphorylated lignin and cellulose nanofibers with the aid of the boric acid as a crosslinker (Zhou et al., 2024). P-O-B bond is formed in the crosslinking network during the combustion. The P-O-B linkage would be transformed into a thermally stable amorphous borophosphate glass. Compared to the phosphate chars, which are easily degraded by the oxidation, the generated protective layer is much more stable with the LOI value of 46% reducing the peak heat release rate to the lowest value of 11.2 W/g. The strong hydrogen bonding between the phosphorylated fibers and boric acid could also improve the cyclic mechanical elasticity of the aerogel, which can be compressed with 90% height recovery, rarely reported in the ceramic-like fire retardant foam (Zhou et al., 2024).

### 6.4 From freeze-drying to scalable foams

While aerogels offer peak thermal insulation performance, their freeze-drying requirement limit their industrial production. An alternative energy-consuming method for drying was recently developed, namely, borate-stabilized foams. By using borates to stiffen the cell walls of pulp suspensions, it is possible to produce functional foam via drying with ambient pressure. Zhang et al. applied a borax-induced self-assembly method to fibers derived from bagasse to produce foams with a porosity of 93.5% and a low thermal conductivity of 63.4 mW/m·K (Zhang X. et al., 2024). The borate ions cross-link the polysaccharides to an extent that pore collapse is prevented during the water evaporation from the foam structure. Bagasse foam was reported to be not only flame retardant, but also hydrophobic with a contact angle of 150.4°, solving the moisture sensitivity problem of cellulosic insulation.

The same approach has been successfully implemented in laminar structures inspired by plant morphology. As shown in Figure 5b, Hou et al. proposed a method based on the use of borates as dual purposes for both flame retardant and binder between cellulose nanofibrils and pulp fibers (Hou et al., 2023). A multi-layer foam with a so-called brick-and-mortar structure was obtained. This laminar structure forces the heat and volatiles to go through a tortuous route within the foam, leading to an enhanced thermal insulation (48.6 mW/m·K) and higher mechanical modulus. Due to the ultra-low density (reported as 12.1 mg/cm3), the boron-mediated assembly is comparable to commercial expanded polystyrene (EPS) with fire resistant. In chitosan foams reinforced by activated carbon and boric acid, a high LOI of 32.9% was obtained with the boron acting as a compatibilizer. The compressive modulus of this material was also enhanced (Ergun, 2023). Polyvinyl acetate (PVAc) foam containing zinc borate and chitosan is another example using the dual actions of boron. While zinc borate is used as a char-forming agent, chitosan acts as a source of carbon, which reduces the mass loss by more than 20% for the foam at 600 °C (Ergun et al., 2023).

### 6.5 Multifunctionality: acoustic and antibiotic properties

Beside the fire resistance, the porous architecture stabilized by boron offer other benefits. For example, zinc borate nanoparticles introduced in carboxymethyl chitosan aerogels improved acoustic absorption coefficient (0.58) while keeping the porosity required to sustain thermal insulation (Li T. et al., 2023). Since borates display biocidal properties, these aerogels and foams are resistant to microbial growth, a key feature in buildings insulation. Wu et al. reported that pulp foams modified with chitosan and cationic polyacrylamide with the borate cross-linking agent has the properties of antibiosis, fire retardancy and sound absorption (Wu et al., 2022).

In summary, for a low-density porous material, boron is not an additive, but an enabler. Researchers used the borate-diol chemistry to construct dynamic self-healing 3D networks and obtain mechanically resilient materials with high porosity with no high flammability. This bio-aerogel and foam is a safer and sustainable alternative to the petroleum-based insulation.

## 7 Discussion and comparative synthesis

Examining how boron-based fire retardancy performs across these disparate material hierarchies, from the macro-scale lignified structure of solid timber to the nano-fibrous networks of aerogels, reveals the contradiction in how boron chemistry is applied. Although the thermodynamic and kinetic mechanisms of boron fire suppression are constant, the methods of embedding these mechanisms into natural fibers differ radically depending on the physical scale of the material and its intended service life. Comparing these categories yields interesting insights regarding the solubility problem, mechanical effects of char promotion, molecular dispersion, and thermal efficiency. A quantitative comparison of these hierarchies is presented in Table 1.

**TABLE 1:** Comparative synthesis of boron-based fire retardancy across material hierarchies.

Material hierarchy	Boron loading strategy	Typical loading (wt%)	Performance (LOI/rating)	Processing intensity
Solid wood	Deep impregnation/Mineralization	High (15%–25%)	30%–45%/V-0 (if mineralized)	Low (vacuum pressure)
Textiles	Sol-gel/Covalent grafting	Low (5%–10%)	28%–35%/Self-extinguishing	Medium (pad-dry-cure)
Composites	Particle filling/Additive	High (20%–40%)	25%–32%/V-1 to V-0	Medium (extrusion)
Aerogels	Structural cross-linker	Low (<10%)	>45%/Non-combustible	High (freeze-drying)

### 7.1 Leaching vs. reversibility

The most enduring challenge of using boron as fire retardant is its water solubility (Obanda et al., 2008). Solubility of boron in water is beneficial to the processing because it allows borates to be transported into the porous structure of wood and textiles without using toxic and volatile organic solvents (Di Blasi et al., 2007; Obanda et al., 2008). Yet solubility is also the ultimate source of failure of boron-based flame retardants in service due to leaching (Kartal et al., 2009; Obanda et al., 2008). The solution to leaching depends on the type of materials.

In wood and bamboo, the strategy to preserve boron is changing from reliance on tortuous cell structures to mineralization (e.g., zinc borate) or entrapment (Camargo and Ibáñez, 2024; Ibáñez et al., 2023; Obanda et al., 2008; Tascioglu et al., 2014; Yu et al., 2017b). In textiles, physical entrapment does not work well because of the large fiber surface area and frequent laundering. Covalent bonds or sol-gel hybrid networks are required to achieve durability of fibers (Chan et al., 2018; Malucelli, 2020; Zhang et al., 2016). In aerogels and hydrogels, where the borate-diol complex is the only cross-linker in the three-dimensional network, the solubility of boron can be important to the leaching of the flame retardant. In addition, solubility is related to the failure of the material (Shang et al., 2017; Spoljaric et al., 2014). Therefore, hydrophobization is necessary not only to maintain fire performance, but also to maintain the aerogel structure when it in contact with water (Shahzadi et al., 2021).

The borates in wood are ionic and highly mobile upon heating, indicating that they can easily flow and seal cracks of wood. Covalently grafted boron moieties are washable, but the mobility of the boron is restricted, which is not favorable for the formation of a glassy continuous barrier over a large surface fissure developed during the early stage of combustion (Chan et al., 2018; Di Blasi et al., 2007; Zhang et al., 2016).

### 7.2 Mechanical integration: additive vs. constitutive roles

For the traditional composites or wood plastic composites, boron minerals are generally considered as fillers with no strength reinforcement. High loadings of zinc borate tend to act as stress concentrators so that the tensile strength of the composite could be reduced and the thermoplastic could be more brittle. Therefore, the fire safety is provided at an expense of the mechanical properties. Thus, coupling agents may be used to help with the compatibility between the incompatible components in the system (Dányádi et al., 2007).

For the bio-based adhesive and aerogel materials, however, the boron, rather than acting as a filler, is an intrinsic component of the system and has a load-bearing role (Alavarse et al., 2022; Li J. et al., 2024; Wicklein et al., 2016). Due to the way boron interacts with the hydroxyl groups, the borate esters act to stiffen and cross-link in the system. As a result, up to a certain saturation level, increasing the amount of boron has no deleterious effect on the modulus or compressive strength of the material. Increasing boron content was found to improve the modulus and compressive strength of the materials. However, the way the boron is introduced into the products may have some effect on the mechanical strength. When boron is added as a discrete filler, there are points of discontinuity in the structure and thus stress transfer is interrupted. When it is added as a chemical cross-linker, it becomes a structural component of the composites so that additional reinforcement is provided. Therefore, if boron can be engineered into the backbone of a polymer, the resulting composites may have the needed fire retardancy without compromising the mechanical strength (Chambhare et al., 2017; Martin et al., 2006).

Whether the boron could catalyze the degradation of the substrate is another consideration. As mentioned previously, boric acid is a Lewis acid, which can catalyze hydrolysis of cellulose chains over time, particularly in a humid environment. This acid catalysis is needed for the formation of the char layer during a fire. If cotton textiles or even paper are stored for a period, they can depolymerize so that their tear strength will decrease (Chen et al., 2025; Stephens et al., 2008; Vibert et al., 2023), referred as tenderizing. When boron species are used in textiles, it is necessary to include buffering agents or complexing ligands so that the acidity of the boron species can be moderated until it is thermally activated (Sricharussin et al., 2004; Yang et al., 2005).

### 7.3 Processing complexity and dispersion limit

A critical trade-off exists between the boron dispersion and the scalability of production. In bulk timber, boron resides as discrete crystalline deposits within the cell lumens rather than within the cell walls. This physical separation introduces a thermal latency. Upon exposure to fire, the retardant first absorbs heat, melt, and flow across the cell wall ready for the fire protection. Conversely, for aerogels and textiles, boron is integrated at the molecular level. This ensures immediate catalytic action with no induction period, yet achieving this level of precision requires complex processing that is currently difficult to scale up (Hassan et al., 2024; Long et al., 2018).

For aerogels and molecularly grafted textiles, the boron atoms are intimate to the cellulosic chains. When heated, a catalytic dehydration takes place instantly (Lin et al., 2024; Malucelli, 2020). Because of this intimate dispersion, that the aerogels can be self-extinguishing with much lower mass loadings compared with timber, where the concentration of retardant is located at the lumen or cellular level. To achieve molecular-level distribution of boron, an energy-intensive process, such as freeze-drying, sol-gel synthesis or layer-by-layer synthesis, must be used, which is difficult for scale-up. It is suggested that the future lies in bringing these molecular efficiencies, preserve of nanoscale, into the scalable processing that is applicable to conventional manufacturing. For example, reactive extrusion can be used to make the composites that exhibit the dynamic cross linking found in hydrogels (Gosecki and Gosecka, 2022).

### 7.4 Synergistic evolution and char stability

It appears that boron alone is rarely the single active element in all modern high-performance systems (Dogan et al., 2021). Boron retardancy refers not only to the evolution of boron chemistry, but also to that of the synergistic combination of boron with other elements, primarily nitrogen and phosphorus. The unique function of boron in these ternary mixtures is to “stabilize” the char (Feng et al., 2015; Zeng et al., 2019). Whereas P is the element responsible for low temperature carbonization and N is the element responsible for the blowing gas of the intumescent process. The carbonaceous char produced by the other two elements is susceptible to afterglow oxidation and to the crumbling of the char skeleton (Feng et al., 2015; Jimenez et al., 2006; Zeng et al., 2019; Zoleta et al., 2020).

Boron compounds bridge the gap between these elements by forming borophosphate or borosilicate glasses which stabilize the char skeleton. This is considered a physical process as the molten glass wets the carbon surface and prevents oxygen from diffusing in. This mechanism is common to all systems whether it is the stabilization of wood char or the stabilization of aerogel nets that crumble easily at high temperatures (Lin et al., 2024; Re et al., 2020). Overall, the ultimate limit of fire protection in the condensed phase for organic materials is to emulate the precursors to ceramic formation using boron to sinter the residual organic residue into a quasi-ceramic state, shifting the charring regime towards ceramization (Song et al., 2019).

The evolution of boron fire retardancy was the adaptation of three fundamental functionalities (acid catalytic charring, glass formation and cross-link formation) to the materials. From using salts to molecularly controlling the interfaces and networks, future work should focus on solving the problem of achieving solubility in solution to ease the synthesis and insolubility in application. Also, the durability of the fire protective action should be improved by replacing the inert fillers with more active structural elements incorporating the boron into the biomaterials.

## 8 Future perspectives and challenges

Based on the above reviews from macroscopic impregnation to molecular level engineering, it spears that the gap between laboratory success and industrial adoption should be bridged given the physicochemical and economical constraints. Instead of pursuing higher Limiting Oxygen Index numbers, future works should focus on resolving the conflicts between fire safety, sustainability, optical aesthetics and processability. Following perspectives are summarized to guide the future boron chemistry that meets these demands.

### 8.1 All-biomass formulations

Current fire-retardant systems are largely based on petrochemical feedstocks. Many studies are on synthetic polymers (e.g., epoxy, polyurethane or melamine formaldehyde) for the matrices or synergistic additives for the boron compounds. The future research lies in all-biomass fire retardants where the boron compounds would pair with naturally derived synergistic additives.

Exploiting inherent chemical functionality of biological macromolecules has gained increasing attention. For example, tannins and lignins are polyphenols containing aromatic structures, which can be converted into char. However, these materials lack thermal stability to withstand ignition temperatures. Boron chemistry provides a viable approach via formation of borate-catechol complexes. A supramolecular system where boron serves as the inorganic node to link the organic precursors, would be one of the future efforts. This approach removes formaldehyde crosslinkers and associated indoor air issues, while impart the flame retardancy. Furthermore, phytic acid, a bio-sourced organophosphate, pairing with boron would provide a renewable P-B system. The key for this approach is to match the molecular weight and crosslinking density to that of synthetic resins for mechanical modulus and water resistance.

### 8.2 Optical transparency and aesthetic preservation

Architectural timber and decorative bamboo are principally valued for the aesthetic quality of the natural grain. Current boron technologies, based on high loadings of zinc borate, or based on the intumescent concept, provide opaque white coatings which hide the substrate. One challenge for future coatings of members required fire retardancy is to provide a high efficiency fire protection treatment which is transparent. This requires a careful control of the phase separation and sizes of boron-containing particles and other nanofiller. Rayleigh scattering requires any dispersed phase to be much smaller than the wavelength of visible light. Therefore, development of boron nano-dispersions or boron incorporated into sol-gels where the refractive index of retardant matches that of the polymer, would be needed. The development of transparent wood (removal of lignin and replacement with polymers with matching refractive index) presents an opportunity for the boron incorporation. Incorporation of boron groups into the impregnating resin at a molecular level will provide an optically clear material at room temperature, which will turn to be opaque with insulation functionality upon heating. The multifunctional material with both optical viability and fire protection upon heating, would be an interesting future development.

### 8.3 Scalability of aerogel

Perhaps the most severe of all scalability challenges exist in the form of nanocellulose aerogels and foams, materials which possess unrivalled thermal insulating properties and excellent flame retardancy characteristics. The production of these materials, historically through freeze-drying or supercritical CO2 drying, relies on water removal at conditions under which surface tension forces do not cause pore collapse; that is, they must be produced under conditions where there is no liquid/gas interface. Both of these drying processes are extremely energy intensive; batch limited and make aerogels unable to compete on cost grounds with cheap mineral wool and expanded polystyrene which dominate the market for thermal insulation.

The industrial future of boron-based foams depends critically on achieving drying at ambient pressure, that is, under conditions when there is a liquid/gas interface. Drying at ambient pressures induces capillary forces as solvent is evaporated from the pores; forces which are generally large enough to crush the fine nanocellulose network (nanocellulose has a skeletal modulus of only a few GPa compared with capillary pressures of several MPa). Boron chemistry could be key to solving this thermodynamic problem. By increasing the density of borate crosslinks that occur between cellulose or alginate chains in the cell walls, the skeletal modulus of the wet gel network can be stiffened and tuned sufficiently to withstand the capillary pressure that is imposed by the drying process. A great deal of future work will need to be dedicated to optimizing these stiffening processes to move towards production of flame retardant foams in continuous conveyor belt reactors. To achieve this, boron-induced stiffening must be integrated with specific chemical strategies for ambient pressure drying, such as surface silylation or solvent exchange, which reduce the surface tension responsible for pore collapse. If the cost of water removal can be decoupled from the production process through these approaches, bio-aerogels may finally take a leap from a lab-confined curiosity to a commodity building material.

### 8.4 Durability and end-of-life circularity

The ultimate long-term environmental fate of the boron treated materials must be analyzed in the context of their life cycle. Although boron-based fire retardants exhibit lower acute toxicity than halogenated analogues, their environmental impact is not negligible. High concentrations of leached boron are phytotoxic, posing risks to soil, and boric acid is classified as a Substance of Very High Concern (SVHC) under EU REACH regulations due to potential reproductive toxicity. Therefore, technologies should be developed that utilize “triggered release” mechanisms where the boron is chemically locked during the service life of the materials to prevent phytotoxic runoff and then recovered during recycling. Unlike thermoset composites, where the polymer matrix is difficult to recycle due to irreversible covalent bonding, boron-crosslinked biopolymers exploit the reversible nature of boron chemistry (boronate esters). This unique chemistry theoretically provides the potential to recycle the boron treated natural fiber composites through a chemical process (i.e., by de-polymerizing the composite under specific pH conditions) so that both natural fibers and boron species are recovered. Should these types of technologies be realized, the circular economy of fire-retardant materials could be fundamentally different than the irreversible covalent treatments—positioning boron as am ultimate solution for fire retardant products considering safety, sustainability, and recyclability.

## 9 Conclusion

Boron-based fire retardancy has gradually evolved from a simple impregnation process to molecular engineering. In the past decades, boron species were used not only as an inorganic additive for fiber-based materials, but also as a key component of biocomposites, bio-adhesives and other bio-products. Due to their versatility, low price, low toxicity and the capability to react with hydroxyl groups, boron species show huge potential to be used in fire safety.

The difference in boron-based fire retardancy across different hierarchies of materials can be clearly observed. For solid timber and flexible textile products, boron species were simply added into the materials through impregnation, layer by layer assembly, or *in situ* polymerization. The fire retardancy originated from the formation of a boron oxide barrier on the surface of the materials, which prevents the diffusion of oxygen and heat transfer to some extent. For bio-adhesives and nanocellulose-based aerogels, boron-based fire retardancy is integrated into the materials where the borate esters are formed between vicinal diols in the cellulose backbone. The boron species play a dual role in adjusting the mechanical rheology of the polymer network and controlling its thermal degradation behavior. The boron-based fire retardancy has been well utilized from the macroscopic level (e.g., solid timber) to the molecular/nanoscopic level (e.g., nanocellulose-based aerogel).

For all applications, the common mechanism is to minimize the production of combustible volatiles by promoting the formation of a stable carbon layer using the boron. Boron, acting as a Lewis acid, modifies the decomposition of cellulose and lignin, promoting dehydration routes that suppress volatilization to levoglucosan. The carbonaceous char is mechanically supported by the formation of glasses (*in situ*, of either borosilicate or borophosphate type), creating a quasi-ceramic residue that withstands high thermal flux. This overcomes the major drawback of organic chars, i.e., their brittleness and oxidative degradation. Boron mineralizes the organic skeleton during the combustion and the resulting material has a greater thermal stability than that of the virgin polymer.

The problem of water solubility is overcome by the sophisticated use of encapsulation and covalently grafted coatings. The development of hydrophobic hybrid coatings and sol-gel organic-inorganic networks allows the water-based boron chemistry to be decoupled from leaching during its service life. This mechanism is particularly applied in textiles and durable composites where supramolecular assembly and synergistic coupling to nitrogen and phosphorus ensure fire resistance during the wetting cycles.

The advancements in boron chemistry allow wide use of natural fiber materials. The link between biological substrate and inorganic fire retardant provided by boron offers renewable, mechanically robust and thermally stable materials. The key future challenge lies in understanding and controlling these interactions, moving towards a closed-loop system. The reversible nature of boron chemistry allows complete recycling of the fire-retardant biocomposites. As the global economy shifts towards sustainable infrastructure, boron-based technologies enable the transformation of the combustible plant matter into safe engineered materials for the built environment.
